# Comparative Genomics of the *Enterobacter cloacae* Mobilome: Prophage Diversity and Anti-Phage Defense Systems in Phage Satellites

**DOI:** 10.3390/v18091045

**Published:** 2026-09-20

**Authors:** Alhassan Alrafaie, Nasser Alqurainy

**Affiliations:** 1 Department of Medical Laboratory, College of Applied Medical Sciences in Al-Kharj, Prince Sattam Bin Abdulaziz University, Al-Kharj 11942, Saudi Arabia; 2 Department of Basic Science, College of Science and Health Professions, King Saud bin Abdulaziz University for Health Sciences, Riyadh 11426, Saudi Arabia; alqurainyn@ksau-hs.edu.sa; 3 Infectious Disease Research Department, King Abdullah International Medical Research Center, Riyadh 11426, Saudi Arabia; 4 Ministry of National Guard-Health Affairs, Riyadh 11426, Saudi Arabia

**Keywords:** *Enterobacter cloacae*, prophages, phage satellites, PICIs, antibiotic resistance, virulence factors

## Abstract

*Enterobacter cloacae* (*E. cloacae*) is an important healthcare-associated pathogen with increasing antimicrobial resistance. Prophages and phage-inducible chromosomal islands (PICIs) are integral components of the *E. cloacae* genome, yet their contribution to resistance, virulence, and anti-phage defense remains poorly understood. To address this gap, we analyzed 35 complete *E. cloacae* genomes, identifying 139 intact prophages (10.8–85.7 kb) with an average GC content (~52%) close to that of the host (54–55%). Most prophages (86.6%) were assigned to the families *Peduoviridae* and *Drexlerviridae* within the class *Caudoviricetes*, and proteome-based clustering showed strong relatedness to phages infecting *Enterobacteriaceae*. The *E. cloacae* genomes harbored multiple antimicrobial resistance genes, notably *fosA* (97%), *oqxA*/*oqxB* (89%) and *blaCMH-3* (77%). However, on genomic-context verification none were genuinely prophage-encoded; they were instead located on the chromosome or plasmids. Virulence profiling revealed universal presence of *ompA* and high prevalence of *csgG* (94%), while the only confirmed prophage-encoded virulence determinants were the Shiga toxin genes *stxA* and *stx1B*. Additionally, 16 phage satellites were identified, containing conserved integration, replication, and packaging modules together with diverse accessory and anti-phage defense systems. Overall, prophages and phage satellites drive genome diversification in *E. cloacae*, with satellites notably serving as reservoirs of anti-phage defense, whereas resistance and virulence genes are located outside prophage and satellite regions.

## 1. Introduction

*Enterobacter cloacae* belong to the *Enterobacter cloacae* complex (ECC) and it is a key opportunistic pathogen in healthcare settings, which causes a variety of hospital-acquired infections including bloodstream, urinary tract, respiratory and wound infections [1,2]. The clinical significance of *E. cloacae* is demonstrated by its rising multi-drug resistance (MDR), which affects therapeutic possibilities and increases risks to public health. ECC isolates show resistance to crucial antibiotics such as carbapenems and cephalosporins and demonstrate the ability to spread between hospitals, which necessitates strong infection control and surveillance systems [3,4]. For instance, carbapenem-nonsusceptible ECC strains carrying antibiotic resistance genes such as bla_NDM-1 bla_IMP-4 and bla_OXA-48 have been associated with outbreaks in intensive care units [2,4,5].

The *E. cloacae* is characterized by its genomic variability due to the acquisition of mobile genetic elements such as prophages integrated into the bacterial chromosome. Prophages play a significant role in bacterial evolution by enabling horizontal gene transfer, increasing genome plasticity and providing adaptive traits which affect both antibiotic resistance and virulence [6,7]. Genes that confer resistance to last-resort antibiotics are identified in prophages, which can lead to MDR outbreaks in healthcare settings [8,9,10]. Prophages also serve as reservoirs for virulence factors that contribute to biofilm formation, immune evasion and increased pathogenicity [11,12,13]. Genomic comparison studies indicate that *E. cloacae* strains have many prophages, with some strains carrying up to a dozen different prophage elements, demonstrating the high prophage diversity in the species [14].

The genomic adaptability of *E. cloacae* is further enhanced by phage satellites. These satellites use helper phages to be excised, replicated and disseminated and therefore enable high-frequency horizontal gene transfer of virulence and resistance determinants [15]. Unlike typical prophages, phage satellites interfere with the phage lytic cycle. This can protect the host populations from phage predation, and satellites may additionally carry adaptive traits, including genes associated with biofilm formation and antibiotic resistance [16].

This study will provide a comprehensive genomic analysis of prophage and phage satellites diversity, antibiotic resistance determinants, and virulence factors in *E. cloacae*, offering new insights into the mechanisms that shape its adaptability and clinical impact. This work addresses important gaps in understanding how *E. cloacae* utilize mobile genetic elements to succeed in nosocomial settings by integrating taxonomic and functional insights into prophage-associated regions and phage satellites.

## 2. Materials and Methods

### 2.1. Bacterial Genome Collection

*Enterobacter cloacae* bacterial genomes were collected from the NCBI Genome database (https://www.ncbi.nlm.nih.gov/datasets/genome/, accessed on 18 May 2024), focusing specifically on complete genomes. A total of 35 genomes were retrieved, and the corresponding GenBank files were obtained for subsequent analyses. These complete genome sequences provided the foundation for prophage identification and further genetic studies. The 35 complete genomes and their GenBank accession numbers are listed in Supplementary Table S1.

### 2.2. Prophage and Phage Satellites Identification

Prophages were identified using the PHASTEST webserver (https://phastest.ca/, accessed on 1 June 2024), which allows for accurate detection of prophage sequences within bacterial genomes. Only intact prophages were selected for further analysis to ensure the reliability of the genetic elements studied. The details of all intact prophages identified can be found in Supplementary Table S1. For each intact prophage, corresponding FASTA files were generated and downloaded for further analyses, including taxonomic classification and functional annotations. For phage satellites, we re-evaluated all prophage regions, specifically those classified as incomplete, questionable, or defective by PHASTEST. To independently validate these findings, we additionally screened each genome using SatelliteFinder v0.9.1 with the publishedMacSyFinder hidden Markov model profiles of Moura de Sousa et al. [17], using default parameters, to identify P4-like elements, phage-inducible chromosomal islands (PICIs), capsid-forming PICIs (cf-PICIs), and phage-inducible chromosomal island-like elements (PLEs), based on hallmark genes and characteristic genomic organization [17]. FASTA files corresponding to these identified satellite regions were extracted for subsequent functional annotation and comparative genomic analyses. Protein-coding sequences for each satellite region were extracted from the matching GenBank annotation and viewed using SnapGene v7.2.1 (GSL Biotech LLC (Dotmatics), San Diego, CA, USA). To confirm these assignments, the deduced protein sequence of each coding sequence was queried against the NCBI non-redundant (nr) protein database using web BLASTP (https://blast.ncbi.nlm.nih.gov/Blast.cgi, accessed on 20 October 2024) (expect threshold 0.05; word size 3; BLOSUM62 matrix; gap costs 11/1; low complexity filtering enabled; maximum target sequences 100). Functional assignments were based on the highest-scoring hits, which were investigated manually and accepted only when the alignment spanned the majority of the query and the annotated function was consistent across multiple independent hits. Family assignments were accepted only where the manual annotation and the SatelliteFinder prediction agreed. Pairwise BLASTN alignment was used to establish the nucleotide identity between the EFN743 and IsolateF cf-PICIs (default settings).

### 2.3. Prophage Taxonomy

The taxonomic classification of each identified prophage was performed using the PHABox webserver (https://phage.ee.cityu.edu.hk, accessed on 5 July 2024), which assigns prophages to their respective families. Phagescope webserver (https://phagescope.deepomics.org/, accessed on 10 July 2024), was also used for Prophage Taxonomy. This process allowed for the identification of common prophage families across the *Enterobacter cloacae* genomes, contributing to the understanding of prophage diversity within these strains.

### 2.4. Antibiotic Resistance Gene Identification

To detect antibiotic resistance genes in both bacterial genomes and prophages, the ABRicate tool (https://usegalaxy.eu/, accessed on 1 August 2024), available on the Galaxy Server, was employed. The ResFinder database was used within ABRicate to identify the presence of antibiotic resistance genes. ABRicate was run with default parameters, applying a minimum threshold of 80% nucleotide identity and 80% coverage. This method provided comprehensive data on the types and frequencies of antibiotic resistance determinants present in the bacterial genomes and prophages.

### 2.5. Virulence Factor Identification

Virulence factors were identified using the Virulence Factor Database (VFDB) through the ABRicate tool (https://usegalaxy.eu/, accessed on 20 August 2024). ABRicate was run with default parameters (minimum 80% identity and 80% coverage), as applied for resistance-gene screening. This analysis aimed to detect potential virulence-associated genes within the prophages and bacterial genomes, contributing to our understanding of the pathogenic potential of *Enterobacter cloacae* strains.

The AMR and virulence genes reported within PHASTEST-predicted prophage regions—the four virulence factors (*fliG*, *astA*, *stxA*, *stx1B*) and the AMR genes of strains ECNIH2 and NH77—were verified by inspecting their flanking genes in the corresponding RefSeq/GenBank-annotated genome for phage structural genes (integrase, terminase, portal, capsid, tail); a gene embedded within such a module was retained as prophage-encoded, otherwise it was assigned to its chromosomal or mobile-element context. This check targeted these specific cargo candidates, not all predicted prophage regions.

### 2.6. Anti-Phage Defenses Identification

Anti-phage defense systems were identified using the DefenseFinder webserver (https://defensefinder.mdmlab.fr/, accessed on 20 September 2024) with default parameters [18]. Nucleotide FASTA files of phage satellite sequences were uploaded to the platform, and DefenseFinder detected known anti-phage mechanisms based on its curated database. Next, all the identified defense genes were annotated directly into the genomic maps for visualization and interpretation. All analyses in this study were descriptive; no formal statistical testing was performed.

## 3. Results

### 3.1. Prophage Identification in Enterobacter Cloacae Strains

**Figure 1:**
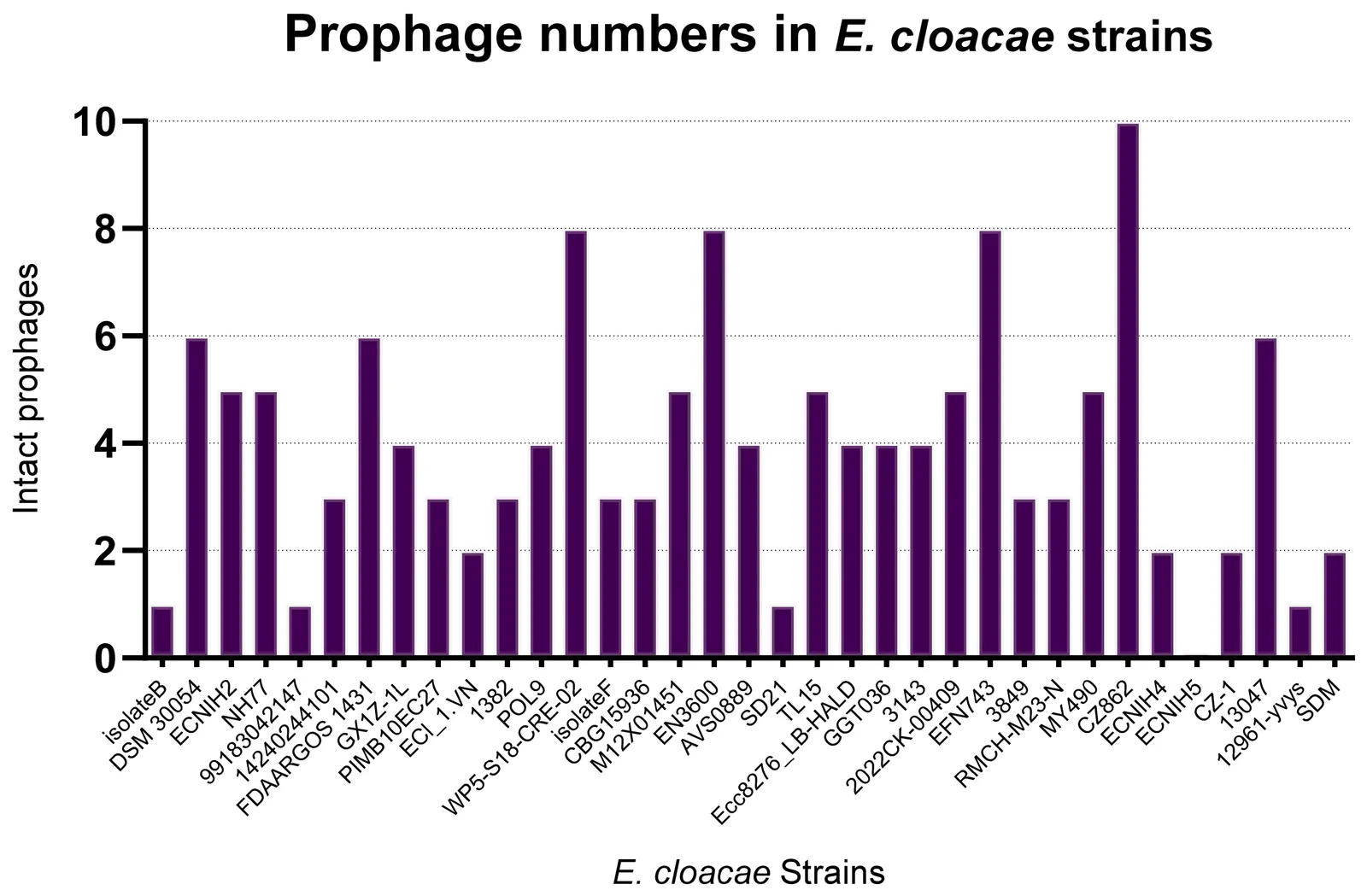
Prophage numbers in *Enterobacter cloacae* strains. The bar chart illustrates the number of intact prophages detected in various *E. cloacae* strains. Each bar represents a specific strain, labeled on the *x*-axis, while the *y*-axis indicates the number of intact prophages identified in that strain.

In our work, we analyzed a total of 35 complete *Enterobacter cloacae* genomes obtained from the NCBI’s bacterial genomes database. A total of 139 intact prophage genomes were identified using the PHASTEST webserver. The number of prophages in each *E. cloacae* genome varied widely from no prophages identified, as in the ECINH5 strain, up to as high as 10 prophages in cz862 strain (Figure 1). Some strains harbored high numbers of prophages, such as EFN743 with 8 prophages, while others contained only a few (1–2), such as IsolateB. Moreover, the size of prophages varied from 10.8 Kb in CZ862 to 85.7 Kb in CBG15936 and encoding 14 and 115 potential protein coding sequences, respectively. Regarding the GC content, the average for the identified prophages was 52% (43.7–56.5%) closely matching the host GC content (54–55%) [19] Supplementary Table S1.

### 3.2. Taxonomy of Prophages

**Figure 2:**
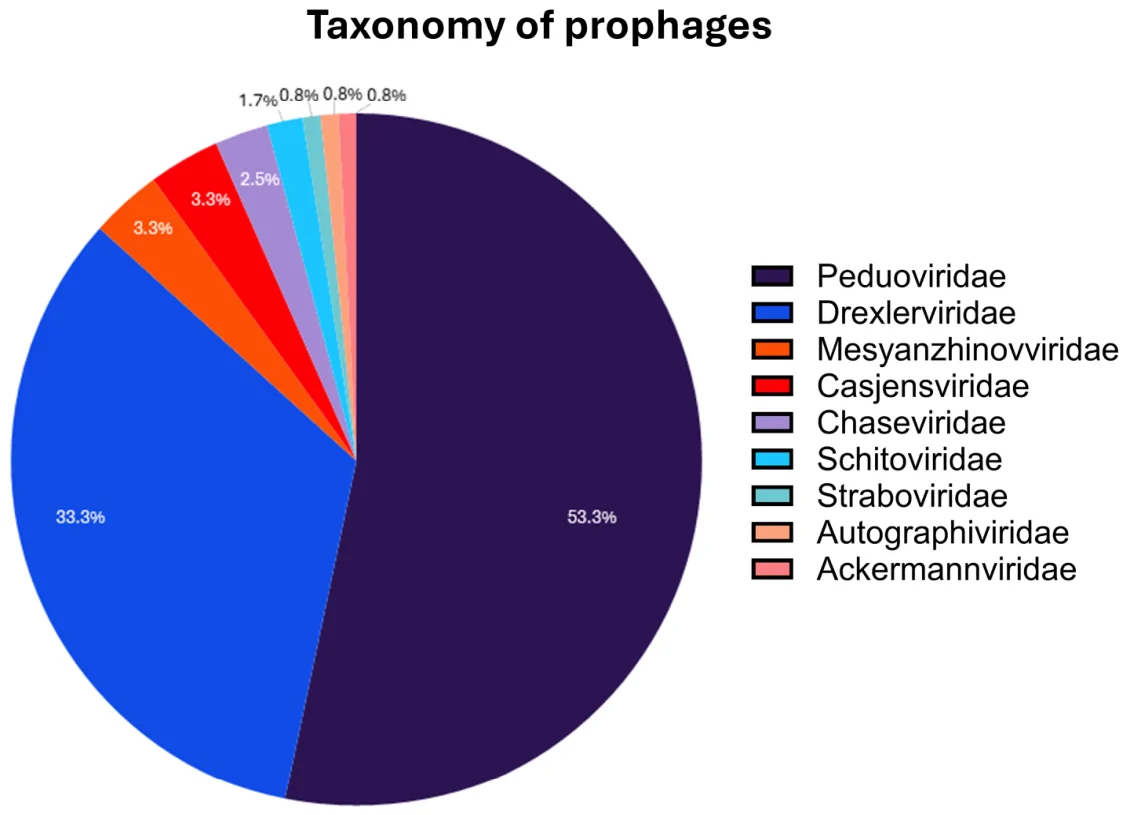
Taxonomy of prophages in *Enterobacter cloacae*. The pie chart depicts the distribution of prophage families identified in *E. cloacae* strains. The largest proportion of prophages belongs to the family *Peduoviridae* (53.3%), followed by *Drexlerviridae* (33.3%). Other families, including *Mesyanzhinovviridae* (3.3%), *Casjensviridae* (3.3%), *Chaseviridae* (2.5%), *Schitoviridae* (1.7%), *Straboviridae* (0.8%), *Autographiviridae* (0.8%), and *Ackermannviridae* (0.8%), contribute to a smaller fraction of the total prophage diversity. Percentages are calculated relative to the 120 prophages assigned to a family and therefore sum to ~100%. The remaining 19 of the 139 prophages could not be assigned to a family; as described in the Results, Phagescope placed these in the class *Caudoviricetes*, with one (CZ862prophage1) in the family *Inoviridae*.

The identified prophages were also analyzed in terms of their taxonomic classification using the Phabox webserver. This analysis revealed that most prophages belong to the families *Peduoviridae* (53.3%) and *Drexlerviridae* (33.3%) (of the 120 prophages that were assigned to a family), indicating their dominance in the *E. cloacae* genomes (Figure 2). Prophages belonging to other families were also identified such as *Mesyanzhinovviridae* (3.3%), *Casjensviridae* (3.3%), and *Chaseviridae* (2.5%). Less than 2% of prophages were assigned to other families such as *Schitoviridae*, *Straboviridae*, *Autographiviridae*, and *Ackermannviridae* demonstrating the broad taxonomic heterogeneity of prophages associated with *E. cloacae*. Using the Phabox webserver, 19 prophage genomes remained taxonomically unclassified, which were then assessed using the Phagescope webserver that assigns viral taxonomy based on 8 taxonomical groups. The Phagescope analysis showed that all analyzed prophages belong to the viral class *Caudoviricetes* except one prophage (CZ862prophage1) which was classified under the family *Inoviridae*, demonstrating the presence of non-tailed filamentous phages in *E. cloacae*.

### 3.3. Proteome-Based Classification of E. Cloacae Prophages

**Figure 3:**
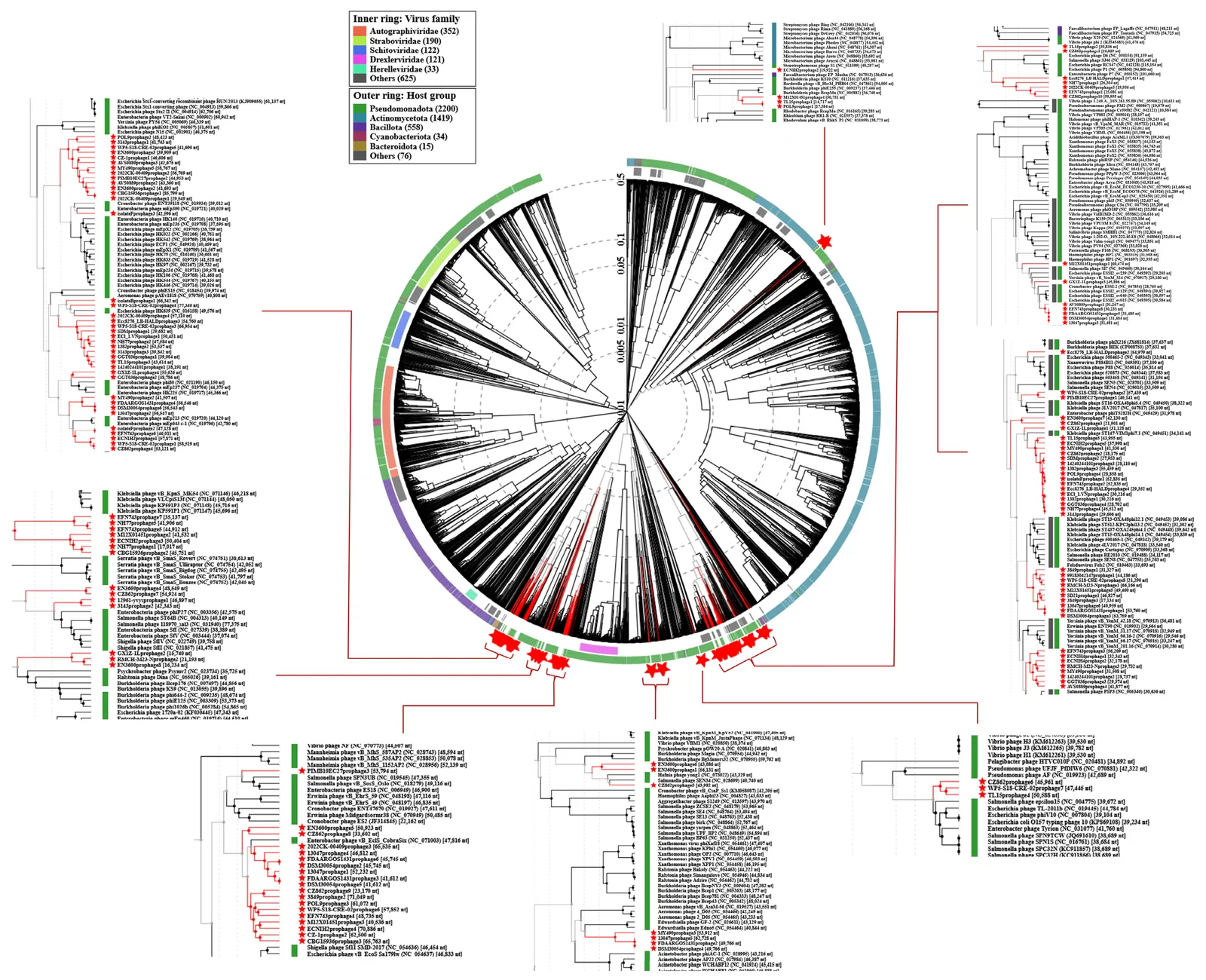
ViPTree genome-wide similarity tree (tBLASTx) of prophages identified in *Enterobacter cloacae* strains (this study) against reference viral genomes. Red stars and red branches mark the study prophages; black branches are reference phages. The inner ring shows virus family (Autographiviridae, red; Straboviridae, light green; Schitoviridae, purple; Drexlerviridae, pink; Herelleviridae, teal; Others, grey) and the outer ring the host group (Pseudomonadota, dark green; Actinomycetota, blue; Bacillota, purple; Cyanobacteriota, olive; Bacteroidota, brown; Others, grey); key numbers are genome counts. Dashed circles are the similarity (SG) distance scale. Boxed panels are magnified clades of the study prophages, each tip labelled with phage name, accession, and genome size.

Our next analysis involved assessing similarities between the identified prophages and other phages publicly available in databases. This was performed using the ViPTree webserver (https://www.genome.jp/viptree, accessed on 1 October 2024), which resulted in some identified *E. cloacae* prophages such as MY490prophage2 and 13047prophage2 showing close similarity to other *Enterobacter* phages like phi80 and HK225 (Figure 3). Other *E. cloacae* prophages such as TL15prophage5 and EFN743prophage8 were found to be related to phages that belong to other *Enterobacteriaceae* members, like *Escherichia* phage ESS12_ev015 and *Klebsiella* phage ST147-VIM1phi7.1. Interestingly, few *E. cloacae* prophages, such as ECNIH2prophage2 and TL15prophage1, showed relatedness to phages infecting hosts outside the *Enterobacteriaceae* family like *Burkholderia* phage BeepMu and *Stenotrophomonas* phage S1. These findings highlight that *E. cloacae* prophages exhibit extensive genetic diversity while showing potential evolutionary relationships between different bacterial families.

### 3.4. Prevalence of AMR Genes in E. Cloacae Strains and Prophages

**Figure 4:**
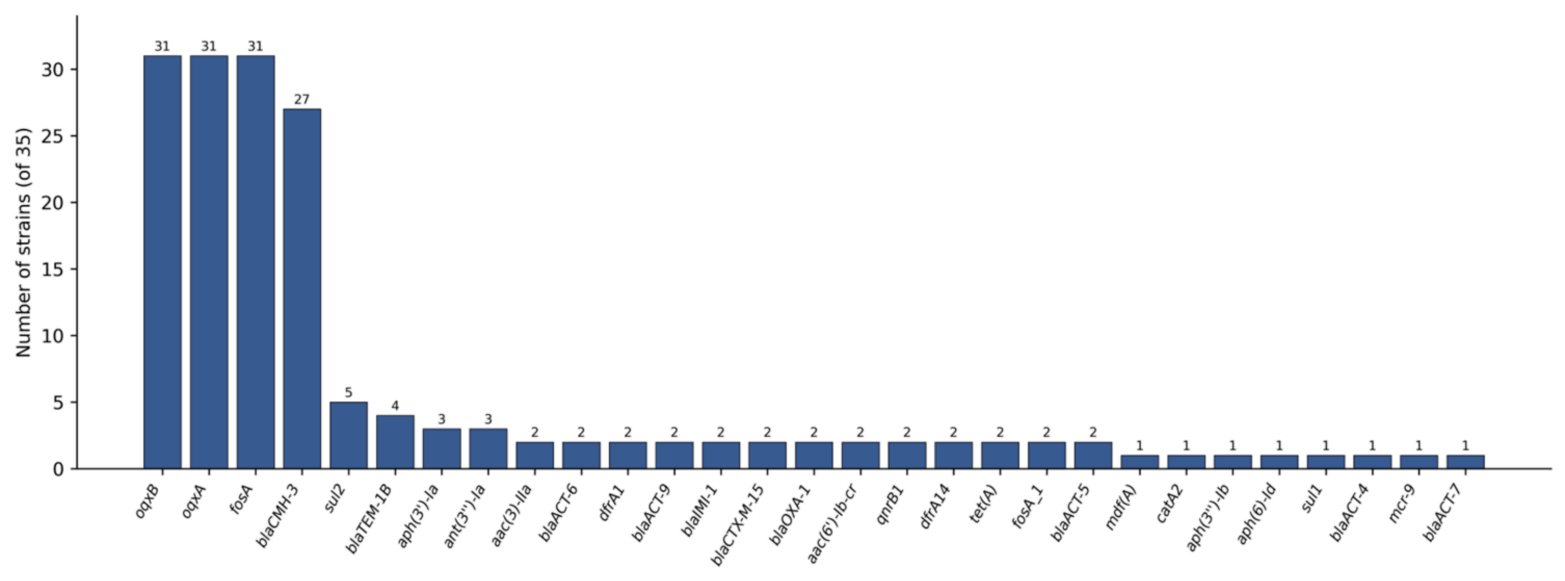
Prevalence of antimicrobial resistance genes across the 35 *Enterobacter cloacae* genomes. Bars show the number of strains (of 35) carrying each AMR gene. All AMR genes were located on the chromosome or plasmids; none were prophage-encoded (see text).

Investigation and understanding of AMR genes is needed to facilitate tackling the AMR crisis. Therefore, our work also analyzed the AMR genes within both the *E. cloacae* strains and identified prophage genomes. For bacterial genomes, some AMR genes (*oqxA*, *oqxB*, and *fosA*) were highly prevalent and identified in 34 of 35 strains for *fosA* (97%) and 31 strains for *oqxA*/*oqxB* (89%) which provide resistance to quinolones and fosfomycin antibiotics [20] (Figure 4). Another identified AMR gene with high prevalence was *blaCMH-3* (identified in 27 strains, 77% of total strains) that contributes to beta-lactam antibiotic resistance [21]. In contrast, other AMR genes were less frequent such as *mcr-9* and *catA2* which provides resistance to colistin and chloramphenicol, respectively [22,23]. It is notable that all analyzed bacterial genomes contain AMR genes, with some having up to 17 varied AMR genes like the clinical isolate EFN743. For EFN743, the identified AMR genes include: *dfrA14_5* (trimethoprim), *qnrB1_1* (quinolones), *aac(3)-IIa_1* (aminoglycosides), *blaOXA-1_1* (beta-lactams), *aac(6′)-Ib-cr_1* (aminoglycosides and fluoroquinolones), *blaCTX-M-15_1* (extended-spectrum beta-lactams), *blaTEM-1B_1* (beta-lactams), *aph(6)-Id_1* (aminoglycosides), *aph(3′′)-Ib_5* (aminoglycosides), *sul2_2* (sulfonamides), *tet(A)_6* (tetracyclines), *oqxA_1* (quinolones), *oqxB_1* (quinolones), *fosA_7* (fosfomycin), *blaCMH-3_1* (beta-lactams), *ant(3′′)-Ia_1* (aminoglycosides), and *dfrA1_8* (trimethoprim). Another example is the clinical isolate CZ862 that Harbored multiple AMR genes: *blaTEM-1B_1* (beta-lactams), *catA2_1* (chloramphenicol), *aph (3′)-Ia_10* (aminoglycosides), *blaCMH-3_1* (beta-lactams), *fosA_7* (fosfomycin), and *sul2_2* (sulfonamides).

For prophage genomes, initial screening flagged AMR determinants within two PHASTEST-predicted regions (strains ECNIH2 and NH77; Figure 4). On genomic-context verification, however, neither corresponded to genuine prophage cargo: both were chromosomal—an intrinsic *fosA* and an integron/IS-associated resistance island misclassified as prophages. We, therefore, found no AMR gene to be genuinely prophage-encoded; resistance determinants in *E. cloacae* are located on the chromosome (including integrative resistance islands) or on plasmids rather than within prophages or satellites.

### 3.5. Prevalence of Virulence Factor Genes in E. Cloacae Strains and Prophages

**Figure 5:**
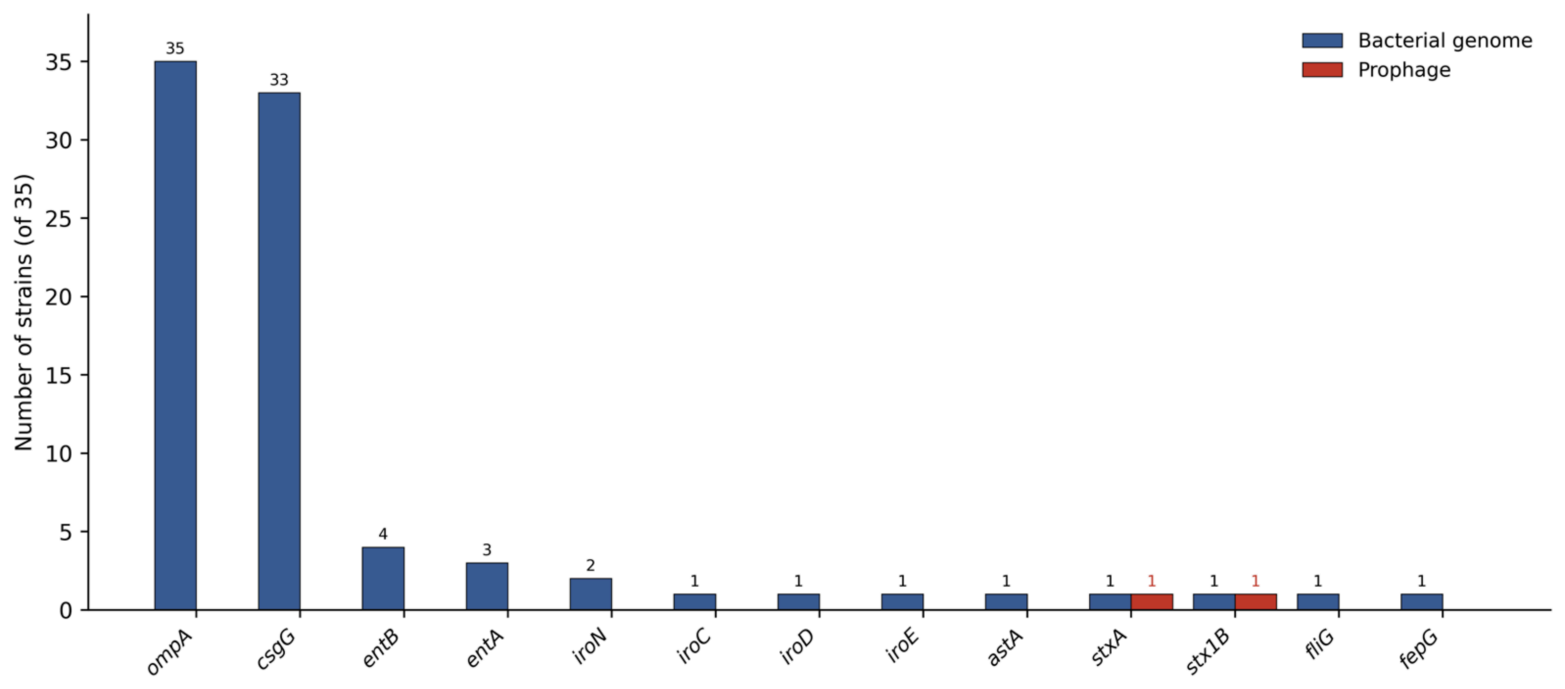
Prevalence of virulence factors across the 35 *Enterobacter cloacae* genomes and their prophages. Blue bars, hits in the bacterial genome; red bars, hits within prophages. Only the Shiga toxin genes (*stxA* and *stx1B*; strain M12 × 01451) were prophage-encoded; *ompA*, *csgG* and the remaining factors are chromosomal.

The analysis of virulence factors in the *E. cloacae* genomes revealed a diverse array of pathogenicity-associated genes. The most prevalent virulence factor was *ompA*, encoding an outer membrane protein involved in immune evasion and adhesion, which was detected in all analyzed genomes (Figure 5) [24]; we note that OmpA also has essential membrane roles beyond pathogenicity [24]. Another prevalent factor was *csgG* (94% of genomes) [25], which facilitates curli-mediated biofilm formation and colonization. Enterobactin-based iron-acquisition genes (*entA*, *entB*), the iron-acquisition system (*iroN*, *iroC*, *iroD*, *iroE*) and the iron transporter *fepG* were less prevalent (genomes ECNIH2 and SDM). Bacterial toxin genes were also detected, most notably the Shiga toxin genes *stxA* and *stx1B*, which cause cellular damage and contribute to severe disease. A hit to *astA* (heat-stable enterotoxin EAST1) was found to correspond to a metabolic arginine N-succinyltransferase and was not retained (Section 3.5).

For prophage genomes, genomic-context verification confirmed only the Shiga toxin genes *stxA* and *stx1B* as genuine prophage-encoded virulence factors, located within a bona fide Stx-converting prophage in strain M12 × 01451. Two other hits initially assigned to prophages (*fliG* and *astA*) were reassigned to chromosomal loci on inspection and were not retained. Shiga toxin genes are classically prophage-encoded, consistent with prophages acting as vehicles for the horizontal spread of specific virulence traits [14]. In contrast, *ompA*, *csgG*, *entA*, and *fepG* were not prophage-associated.

### 3.6. Identification and Characterization of Phage Satellites in Enterobacter Cloacae

A total of 16 phage satellites were identified within 15 of the 35 analyzed genomes. Their genomic size ranged from approximately 11.1 kb to 18.5 kb [17], and they are distributed across different strains from various sources, including clinical, environmental, and wastewater. The identified satellite elements were divided into three distinct categories: P4-like elements, PICIs, and cf-PICIs, based on gene content and phage-exploitation modules (Table 1). No PLEs were detected, a fourth family screened by SatelliteFinder [17]. Each category is determined by a characteristic combination of core functions, including integration, regulation, replication, and phage capsid hijacking, as detailed in Table 1. The P4-like family was the largest group, accounting for eight elements (50%), followed by six classified as classical PICIs (37.5%) and two as cf-PICIs (12.5%).

All 16 satellites were integrated into the bacterial chromosome via a site-specific integrase. One strain, NH77, carried two distinct elements—a P4-like element and a classical PICI—within a single host chromosome; the remaining 14 strains each carried one.

### 3.7. P4-like Elements

**Figure 6:**
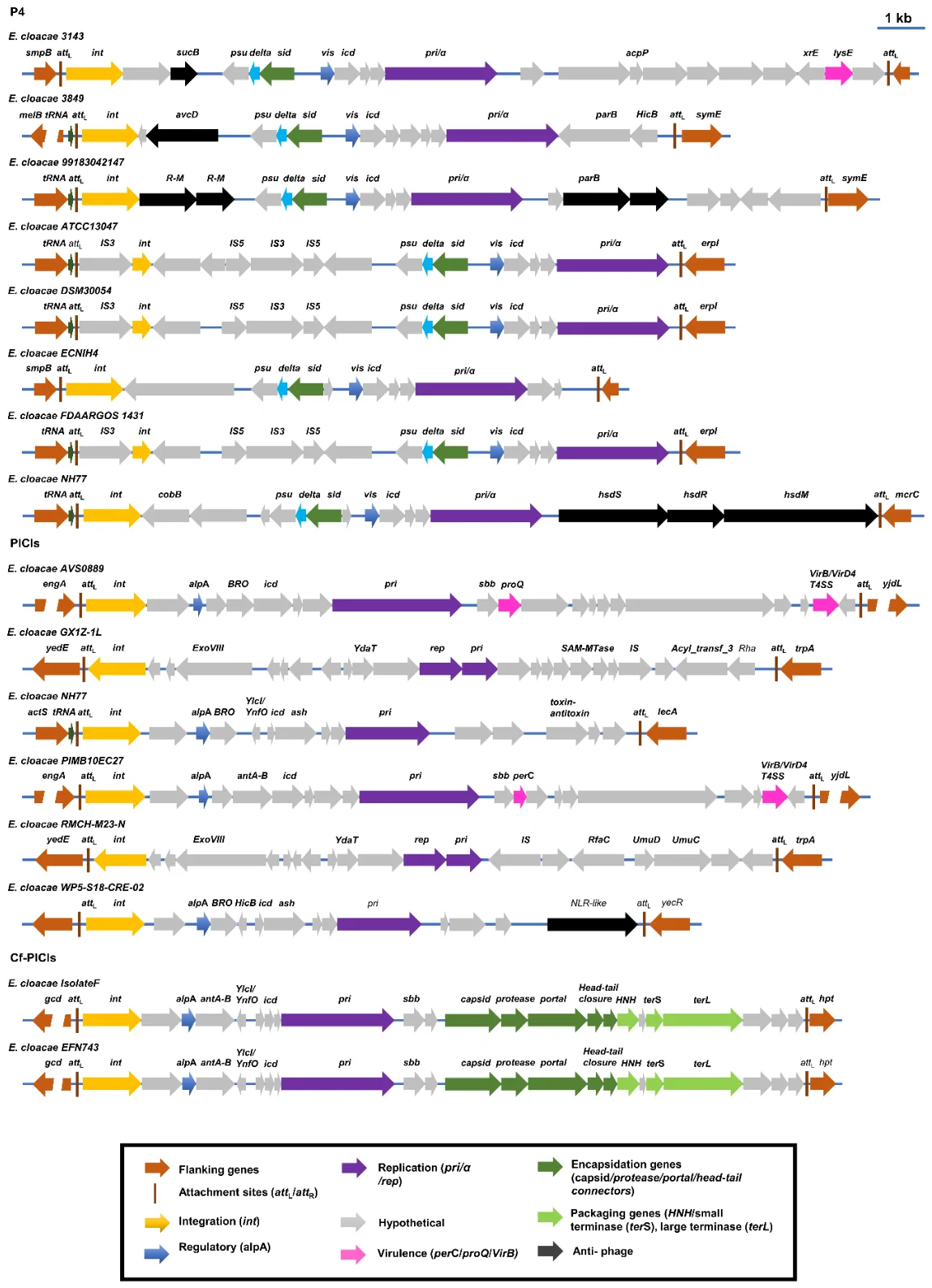
Comparative genomic architecture of *Enterobacter cloacae* phage satellites. Genomic maps of all 16 satellite elements identified in this study, grouped by lineage (eight P4-like elements, six classical PICIs, and two cf-PICIs). Maps are aligned according to the prophage convention, with the integrase gene (int) at the left end; scale bar, 1 kb. Genes are colored by functional module: integration (int), yellow; attachment sites (attL/attR); transcriptional regulators (alpA), dark blue; replication genes (pri/α, rep), purple; encapsidation genes (capsid, protease, portal, head–tail connector), green; packaging genes (HNH, small terminase [terS], large terminase [terL]), light green; virulence-associated regulators and host-interaction genes (perC, proQ, VirB/VirD4), pink; putative anti-phage defense systems, black; hypothetical proteins, gray; flanking chromosomal genes, brown.

We identified eight compact P4-like satellite elements, ranging in size from 11.1 to 18.5 kb, within the analyzed *E. cloacae* strains (3143, 3849, 99183042147, ATCC 13047, DSM30054, ECNIH4, FDAARGOS1431, and NH77) (Figure 6). As shown in the genomic maps, these elements encode a minimal gene set characteristic of the P4 satellite family [25]. All the eight elements contain a site-specific integrase (*int*) together with Sid, Delta, and Psu, predicted to mediate hijacking of the helper-phage capsid; a regulatory protein annotated as Vis, a known AlpA homolog [26]; and a primase/α replication protein.

### 3.8. Classical PICIs and Capsid-Forming PICIs (cf-PICIs)

Six phage satellites (11.4–16.8 kb) that align with the canonical architecture of Gram-negative PICIs were identified, in strains AVS0889, GX1Z-1L, NH77, PIMB10EC27, RMCH-M23-N, and WP5-S18 (Figure 6). All six encoded the standard PICI core module: a site-specific integrase, a replication initiator (a primase, typically with an origin of replication), and a master regulator of the AlpA family in four elements (AVS0889, NH77, PIMB10EC27, WP5-S18) and an excisionase in the remaining two (GX1Z-1L, RMCH-M23-N). Consistent with the classical PICI architecture, all six lacked the large terminase (TerL), portal, and other major structural genes, and are therefore predicted to rely on the helper phage for encapsidation [27,28]. In addition, two of the classical PICIs (NH77 and WP5-S18-CRE-02) encoded a homolog of ash, which is a gene first described in P4 that inactivates the helper-phage repressor and thereby triggers its induction [29].

Two cf-PICIs were identified, in strains EFN743 and IsolateF, which share 99.99% nucleotide identity (100% query coverage) and therefore represent a single cf-PICI genotype present in two isolates rather than two independent elements. In contrast to the classical PICIs, both encoded an expanded structural repertoire comprising a major capsid protein, portal, protease, head–tail connector, TerS, and the large terminase subunit (TerL) (Figure 6). Tail-morphogenesis and host-lysis genes were not identified in either element.

### 3.9. Phage Satellites Encode Diverse Anti-Phage Systems and Accessory Cargo

Across the 16 satellites, the core genes were conserved whereas the accessory regions were variable and encoded genes associated with phage defense, virulence, and host adaptation (Table 1). DefenseFinder identified six putative anti-phage systems in five satellites (31.3%). These include a Type I restriction–modification (R–M) system (HsdR, HsdM, HsdS) in the P4-like elements NH77; a Type II R–M system together with a PD-Lambda-5 system in 99183042147 [30]; a Sucellos system (*sucB*) in 3143 [31]; a dCTP deaminase (*avcD*) in 3849, predicted to deplete deoxycytidine nucleotides during infection and thereby limit phage replication [32,33]; and an NLR-like bNACHT protein in the PICI WP5-S18-CRE-02 [34].

Additional accessory genes, including regulators, toxin–antitoxin modules, and metabolic enzymes, were identified (Table 1). An Icd-like protein was present in most elements. The PICI PIMB10EC27 encodes a putative PerC-family transcriptional regulator, and AVS0889 encodes a putative ProQ/FinO-like RNA chaperone. Putative phage antirepressors of the BRO, AntA/AntB, and Rha families were detected across multiple elements, as were toxin–antitoxin modules (HicB, YdaT-like) (Table 1). The P4-like element in 3143 encoded a long-chain fatty acid CoA ligase and a LysE family transporter; the PICIs in PIMB10EC27 and AVS0889 encoded VirB/VirD4 Type IV secretion system (T4SS) components; RMCH-M23-N carries a predicted *umu*DC mutagenesis cassette; and GX1Z-1L encoded a SAM-dependent methyltransferase.

In addition, we identified mobile genetic elements, specifically insertion sequences (IS) or transposases, in 5 of the 16 satellites (31.3%). These included members of the IS3, IS5, and IS903B families, prominently detected in the P4-like satellites of strains DSM30054, FDAARGOS 1431, and ATCC13047, as well as an IS1-like (IS1B family) element in the PICI of GX1Z-1L and an IS3 element in the PICI of RMCH-M23-N. All reported gene functions are based on sequence homology and were not experimentally validated.

## 4. Discussion

The *Enterobacteriaceae* family contains *Enterobacter cloacae* as a clinically important member which has become increasingly difficult to treat with antibiotics due to its flexible nature and antibiotic resistance [3]. *E. cloacae* adapts its evolutionary behavior through the utilization of prophages as genetic diversity drivers which facilitate horizontal gene transfer (HGT) [14]. This study analyzed 35 *E. cloacae* strains that contained 139 intact prophages which spanned from none in ECINH5 to ten in CZ862 strains and had genome sizes ranging between 10.8 and 85.7 kb. The diversity observed confirms that *E. cloacae* possess diverse prophages like other bacteria and *Enterobacteriaceae* members [35,36,37].

The smallest identified prophage contained 10.8 kilobases, possessed 14 CDS, and falls into the small phage genomes [38]. In contrast, the largest prophage genome of 85.7 kb contained 115 CDS which confer numerous functional genes that could enhance the survival capabilities and adaptability of the host. Additionally, the genetic GC content between prophages (average 52% GC content) and their host chromosomes (54% GC content) is consistent with long-term residence and progressive amelioration of prophage sequences toward the host base composition, although the lower GC values of some prophages may reflect more recent acquisition [39]. The genomic compatibility between host bacteria and their prophages enables the preservation of these elements while they promote gene exchange and structural variations in the genome.

The taxonomic profiling of *E. cloacae* prophages showed that *Peduoviridae* and *Drexlerviridae* were the most prevalent families, as reported in previous studies on *Enterobacteriaceae* phages [40,41]. Less frequently, prophages from *Casjensviridae*, *Chaseviridae* and filamentous *Inoviridae* families were identified showing that *E. cloacae* genomes contain a wide range of phages [42]. The diverse phage population indicates a complex history of phage exposure and possible interspecies phage transfer which reflects the dynamic interactions between *E. cloacae* and its viral predators [43]. The use of Phabox and Phagescope web tools emphasized the classification results confirming the prevalence of phage class *Caudoviricetes* while showing occasional integration of non-tailed filamentous.

Genome-wide similarity analysis using ViPTree provided further insight into the sequence relatedness of *E. cloacae* prophages. The prophages showed high similarity to well-characterized *Enterobacter* phages such as phi80 and HK225, consistent with a shared evolutionary history within the genus. Several prophages also shared sequence similarity with phages infecting other *Enterobacteriaceae* and, in some cases, more distant genera such as *Burkholderia* and *Stenotrophomonas*. Importantly, ViPTree reflects genome-wide sequence and proteomic similarity and does not, by itself, establish the timing or direction of gene transfer; these patterns therefore indicate potential evolutionary connectivity rather than demonstrated recent horizontal gene transfer.

Beyond autonomous prophages, our analysis revealed that this viral landscape is further shaped by the presence of 16 phage satellites, which we classified into three distinct lineages: P4-like elements, classical PICIs, and capsid-forming PICIs (cf-PICIs). P4-like elements were the largest group (50%), consistent with their widespread distribution in *Enterobacteriaceae* [44]. No PLEs were detected, which is expected given that this family has so far been described only in *Vibrio cholerae* and its virulent helper phage ICP1 [45]. All three satellites rely on helper phage, which defines phage satellites [16], but they differ in the extent of that dependence. Classical PICIs hijack the helper capsid outright [27], The cf-PICIs, in contrast, encode a complete capsid and packaging module, including the large terminase (TerL). Their dependence on the helper phage is therefore restricted to induction and tail morphogenesis and lysis, positioning them as an evolutionary intermediate between classical satellites and autonomous phages [46]. The near-identity of the two cf-PICIs recovered here from different isolates is consistent with recent evidence that cf-PICI capsids can be released without tails and acquire tails from unrelated phages, broadening the range of strains they can reach [47].

The coexistence of two satellites in the strains NH77, P4-like, and PICIs is notable. Because the two belong to different satellites and are likely to depend on different helper phages, direct competition between them may be limited. We hypothesize that their regulators respond to distinct inducing signals, ensuring that each element mobilizes only with its cognate helper; this remains to be tested experimentally.

Regarding resistance, the *E. cloacae* strains contained various prevalent antibiotic resistance (AMR) genes such as *oqxA*, *oqxB* (quinolone resistance), *fosA* (fosfomycin resistance) and *blaCMH-3* (beta-lactam resistance) that align with worldwide patterns of multi-drug resistant *Enterobacteriaceae* [48,49]. All strains maintained at least one AMR gene and the EFN743 clinical isolate displayed the highest number of 17 different AMR genes leading to resistance against various antibiotic classes. This creates a major clinical hazard mainly in hospital environments where MDR strains restrict available treatments while elevating patient mortality rates. The detection of various AMR genes in clinical isolates emphasizes the immediate requirement for enhanced surveillance programs and infection control practices. For prophages, a low number of AMR genes was identified within their regions (1.4%) and none were found in the satellite elements. This matches previous findings which show that AMR genes mostly reside on plasmids not prophages or satellite genomes [50].

The virulence factor (VF) analysis showed *ompA* as the dominant VF, present in every analyzed genome and essential for adhesion, immune evasion and biofilm formation across Gram-negative bacteria [24]; it also has essential membrane functions beyond pathogenicity [24]. The VF *csgG* was likewise prevalent among *E. cloacae* strains and contributes to curli fimbriae and biofilm maturation [51]. Iron-acquisition systems (*entA*, *entB*, *iroN*, *iroC*, *iroD*, *iroE*) were detected less frequently and supported survival in low-iron host environments [52]. Following genomic-context verification, the Shiga toxin genes (*stxA*, *stx1B*) were the only confirmed prophage-encoded virulence factors, consistent with the established role of Stx-converting prophages in disseminating this toxin.

An important outcome of our integrative analysis was the ability to separate genes genuinely carried by mobile elements from host genes captured within predicted prophage boundaries. On re-examination of genomic context, several determinants initially detected within predicted prophage regions were reassigned to chromosomal loci. These cases illustrate a broader caveat for mobilome surveys that rely on automated prophage prediction: boundary calls can incorporate flanking chromosomal genes, and virulence or resistance databases can contain gene-name ambiguities, so cargo assignments are most robust when corroborated by inspection of the surrounding gene neighborhood. Applying this criterion refined rather than weakened our conclusions, and we recommend it as a routine step in comparable analyses.

The one virulence determinant that could be confidently attributed to a prophage was the Shiga toxin operon (*stxA*–*stx1B*), which lay within a complete, integrase-anchored prophage in strain M12X01451 and was flanked by canonical phage genes. Stx-converting lambdoid prophages are the classical vehicle for Shiga toxin dissemination in *Escherichia coli* and *Shigella*, in which toxin expression is coupled to prophage induction [53]. The presence of a comparable Stx prophage in *E. cloacae* therefore represents a genomic association consistent with the potential for phage-mediated spread of this trait across *Enterobacteriaceae*, rather than evidence of transfer into this species. Although observed in only a single strain here, the presence of a bona fide Stx prophage in an opportunistic nosocomial pathogen is clinically relevant and supports continued surveillance of the toxigenic potential of *E. cloacae*.

Considered together, these observations point to a division of labor among the mobile genetic elements of *E. cloacae*. Resistance determinants were not prophage- or satellite-borne but resided on the chromosome and its integrated resistance islands or on plasmids, consistent with plasmid-, integron- and insertion-sequence-driven mobilization of antimicrobial resistance in *Enterobacteriaceae*. Prophages contributed only sporadic virulence cargo, epitomized by the Shiga toxin operon, whereas phage satellites were enriched instead for anti-phage defense and host-adaptive functions. This partitioning suggests that the different classes of element occupy distinct adaptive niches, and that the clinical importance of prophages and satellites in *E. cloacae* lies less in the spread of resistance than in virulence conversion and in the modulation of phage–host interactions.

In contrast to the prophages, the identified phage satellites may act as defense islands [54], carrying six putative anti-phage systems, a Sucellos system, a dCTP deaminase, Type I and Type II R–M systems, PD-Lambda-5, and an NLR-like bNACHT protein, across five of the 16 elements (31.3%). In our dataset, defense was more concentrated in the P4-like lineage with four out of eight elements encoding at least one predicted system compared with one of six PICIs and neither of cf-PICI. Their presence suggests that satellites may contribute to host immunity against competing phages, potentially benefiting the bacterial population; the activity of these systems was not tested here [54]. This may appear counter-intuitive, since satellites rely on a helper phage for mobilization [16]. Experimentally, however, PICI-encoded defense systems provide broad immunity against phages, plasmid and non-cognate PICIs, and the helper phage that spread these systems gain an advantage over competitors for the same host. Thus, the cost to the helper phage is outweighed by the competitive advantage gained which makes the interaction mutualistic rather than parasitic [55,56].

Beyond defense, the accessory regions encode regulators and enzymes that may influence host physiology, such as the PerC-family transcriptional activator associated with virulence gene expression [57], a ProQ/FinO-like RNA chaperone [58], and a long-chain fatty acid–CoA ligase. The VirB/VirD4 T4SS components carried by the PICIs of PIMB10EC27 and AVS0889 point to possible conjugative mobility; because a complete T4SS is atypical of canonical PICIs, these two elements may represent PICI–conjugative-element hybrids [59]. Collectively, these findings suggest a functional complementarity within the mobilome, where prophages contribute directly to invasion via toxins and motility, while satellites facilitate long-term persistence through host adaptation and defense against viral predation. Insertion sequences of the IS3 and IS5 families flanking accessory regions of approximately one-third of the satellites (5 of 16). These insertion sequences facilitate the acquisition of host-adaptive genes through transposition or homologous recombination, functioning as active ‘hotspots’ for functional diversity [60], and may allow *E. cloacae* to rapidly refine its accessory cargo in response to environmental pressures, such as phage predation or antibiotic exposure, significantly enhancing genomic flexibility.

This study has several strengths and limitations that need consideration. A major strength is the integrative genomic approach, which examined prophages, phage satellites, AMR genes, virulence factors, and anti-phage defense systems from the same *E. cloacae* genome dataset. This strategy enabled us to identify chromosomal resistance determinants that could be distinguished from the viral and satellite regions, which instead carried defense and virulence traits. Although phage satellites have previously been cataloged across bacterial genomes at scale [17], the systematic classification of P4-like satellites, classical PICIs, and cf-PICIs presented here, together with the characterization of their accessory cargo, provides a species-focused view of satellite distribution and function in *E. cloacae*. However, the study is limited by reliance on publicly available genomes and in silico predictions, without experimental validation of prophage induction, satellite mobilization, or functional gene expression. In particular, helper phages were not identified for the satellites described here, and the activity of the predicted anti-phage systems was not assessed experimentally. The dataset may also not fully capture the global genetic diversity of *E. cloacae,* and the accompanying clinical information for many isolates was incomplete or inconsistently reported. Future investigations should prioritize monitoring of *E. cloacae* in clinical environments and the systematic isolation of phages to determine how mobile genetic elements influence strain persistence, transmission patterns, and susceptibility to phage-based therapies.

## 5. Conclusions

This study defines the mobilome landscape of *Enterobacter cloacae* and indicates that it is shaped by a dynamic genomic interplay between the bacterial host, integrated prophages, and phage satellite elements. Our analyses show that antimicrobial resistance determinants are predominantly encoded within the host chromosome, whereas prophages and phage satellites harbor a limited but notable set of virulence-associated genes and a broader repertoire of anti-phage defense-related genomic features. The identification of diverse prophage families alongside P4-like elements, classical PICIs, and capsid-forming PICIs underscores the complexity of viral–bacterial interactions within *E. cloacae*. Overall, these findings show that considering the associated virosphere helps explain how mobile genetic elements shape the genome and clinical significance of *E. cloacae*.

## Figures and Tables

**Table 1:** Genomic characteristics of phage satellites identified in *Enterobacter cloacae* genomes. Genetic architecture of the 16 satellite elements identified in this study, classified into three evolutionary lineages: P4-like elements (characterized by the int-psu-delta-sid capsid-hijacking module), classical phage-inducible chromosomal islands (PICIs) (int-alpA-pri, without structural genes), and capsid-forming PICIs (cf-PICIs) (int-alpA-pri plus encapsidation and packaging modules). Each element’s genomic coordinates are provided with its accession number in parentheses. Core and accessory genes were assigned from the GenBank annotation and confirmed as describe in Section 2.2; anti-phage systems were predicted with DefenseFinder. Abbreviations: int, integrase; alpA, AlpA-family transcriptional regulator; vis, P4 excisionase; pri/primase, replication initiator; rep, replication protein; sid, capsid size-determination protein; delta (δ), late-gene transactivator; psu, polarity suppression/capsid decoration protein; ash, helper-prophage derepressor; TerS/TerL, small and large terminase subunits; icd, Icd-like protein; acpP, acyl carrier protein; CoA ligase, long-chain fatty acid–CoA ligase; lysE, LysE-family efflux transporter; XRE, Xre-family HTH transcriptional regulator; parB, partitioning protein; cobB, sirtuin-family deacetylase; BRO, Bro-N-domain phage regulator; AntA/AntB and Rha, phage antirepressors; HicB, antitoxin of the HicAB toxin–antitoxin system; YdaT, prophage-encoded toxin/regulator; YlcI/YnfO, prophage-associated protein; ProQ, ProQ/FinO-family RNA chaperone; PerC, PerC-family virulence regulator; VirB/VirD4, type IV secretion system components; ExoVIII, RecE-like exonuclease VIII; RfaC, LPS heptosyltransferase; UmuD/UmuC, SOS mutagenesis cassette; Acyl_transf_3, acyltransferase-3 family protein; SAM-MTase, S-adenosylmethionine-dependent methyltransferase; sucB, Sucellos anti-phage system; avcD, dCTP deaminase; HsdR/HsdM/HsdS, type I restriction–modification subunits; RM, restriction–modification; REase, restriction endonuclease; MTase, methyltransferase; PD-Lambda-5, phage-defense system; bNACHT, bacterial NACHT-domain protein; IS, insertion sequence. All the gene functions are predicted from sequence homology and were not experimentally validated. –, not detected.

Satellites	Strains	Accession Number	Size	Core Genes	Accessory Genes	Anti-Phage
Type		(Genomic Location)	(Kb)	(BlastP)	(BlastP)	
P4-like						
	3143	NZ_CP103611	18.5	Integrase, psu, Delta, sid, vis, primase	Icd, acpP, CoA ligase, XRE, lysE	sucB
		(3,727,960–3,746,488)				
	3849	CP052870	12.4	Integrase, psu, Delta, sid, vis, primase	Icd, parB, HicB	avcD
		(804,404–816,859)				
	99183042147	CP134674	17	Integrase, psu, Delta, sid, vis, primase	Icd, parB	RM Type II system (MTases and REases)
		(1,078,566–1,095,528)				PD-Lambda-5 (PD-Lambda-5_A and _B)
	ATCC13047	NC_014121	13.6	Integrase, psu, Delta, sid, vis, primase	IS3 transposase, IS5 transposase, IS3 transposase	–
		(682,057–695,652)			IS5-like IS903B transposase, Icd	
	DSM30054	NZ_CP056776	14.4	Integrase, psu, Delta, sid, vis, primase	IS3 transposase, IS5 transposase, IS3 transposase	–
		(1,088,861–1,103,272)			IS5-like IS903B transposase, Icd	
	ECNIH4	CP009850	11.1	Integrase, psu, Delta, sid, vis, primase	Icd	–
		(1,150,384–1,161,489)				
	FDAARGOS 1431	NZ_CP077211	13.6	Integrase, psu, Delta, sid, vis, primase	IS3-like ISSen4 transposase, IS5 transposase, IS3 transposase	–
		(404,967–418,590)			IS5-like IS903B transposase, Icd	
	NH77	CP040827	17.8	Integrase, psu, Delta, sid, vis, primase	cobB, Icd	RM Type I system (HsdS, HsdR, HsdM)
		(2,166,943–2,149,129)				
PICIs						
	AVS0889	NZ_CP092042	16.8	Integrase, AlpA, primase	BRO protein, Icd, ProQ, VirB/VirD4 T4SS	–
		(3,601,239–3,618,028)				
	GX1Z-1L	NZ_CP071861	14.6	Integrase, excisionase, rep/primase	ExoVIII, Toxin YdaT protein, Class I SAM-Mtase	–
		(3,729,682–3,744,279)			IS1-like element IS1B family transposase, Acyl_transf_3, Rha	
	NH77	CP040827	11.9	Integrase, AlpA, primase	BRO family protein, Icd, ash, Toxin–antitoxin (TA)	–
		(3,799,225–3,811,096)				
	PIMB10EC27	NZ_CP020089	16.5	Integrase, AlpA, primase	AntA/AntB protein, Icd, PerC, VirB/VirD4 T4SS	–
		(3,772,828–3,789,339)				
	RMCH-M23-N	CP123598	16	Integrase, excisionase, Primase	Exodeoxyribonuclease VIII, YdaT family protein	–
		(3,920,333–3,936,315)			RfaC, UmuD, UmuC, IS3	
	WP5-S18-CRE-02	NZ_AP022126	11.4	Integrase, AlpA, Primase	BRO protein, Toxin–antitoxin system HicB, Icd, ash	NLR_like_bNACHT01 system
		(4,161,988–4,173,384)				
cf-PICIs						
	EFN743	NZ_CP092635	16.4	Integrase, AlpA, primase,	AntA/AntB antirepressor, YlcI/YnfO family protein, Icd	–
		(4,387,072–4,403,477)		capsid, portal, protease, head–tail connector, TerS, TerL		
	IsolateF	CP135475	16.4	Integrase, AlpA, primase	AntA/AntB antirepressor, YlcI/YnfO family protein, Icd	–
		(4,177,763–4,194,170)		capsid, portal, protease, head–tail connector, TerS, TerL		

## Data Availability

The datasets analyzed for this study are publicly available in the NCBI GenBank database (https://www.ncbi.nlm.nih.gov/). The accession numbers of the 35 complete *Enterobacter cloacae* genomes analyzed are provided in Supplementary Table S1, and the identified prophage and phage-satellite sequences are included in the Supplementary Material.

## Supplementary Materials

The following supporting information can be downloaded at: https://www.mdpi.com/article/10.3390/v18091045/s1, Supplementary Table S1. Prophages identified in the 35 *Enterobacter cloacae* genomes. Strain, host strain designation; Source, isolation source; Intact prophage, index of each PHASTEST-predicted intact prophage within the strain; Total prophages, number of intact prophages per strain; Size (kb), prophage length; CDS, number of predicted coding sequences; GC%, guanine–cytosine content; Region position, genomic coordinates of the prophage; Taxonomy, viral family assigned by PhaBox/Phagescope; VF, virulence-factor hits (VFDB) within the predicted prophage region; Anti-CRISPR, predicted anti-CRISPR proteins; AMR in prophages, antimicrobial-resistance genes detected within the predicted prophage region; AMR in bacterial genome, antimicrobial-resistance genes located elsewhere on the chromosome or plasmids. All gene assignments are predicted from sequence homology (VFDB, ResFinder/ABRicate, DefenseFinder) and were not experimentally validated; database hits falling within predicted prophage regions were confirmed by genomic-context inspection (see main text).
